# Relationships Between Diagnosis, Bacterial Isolation, and Antibiotic Prescription in Out Patients With Respiratory Tract Infection Symptoms in Rural Anhui, China

**DOI:** 10.3389/fpubh.2022.810348

**Published:** 2022-02-09

**Authors:** Shen Xingrong, Feng Rui, Chai Jing, Cheng Jing, Isabel Oliver, Helen Lambert, Debin Wang

**Affiliations:** ^1^School of Health Service Management, Anhui Medical University, Hefei, China; ^2^Department of Literature Review and Analysis, Library of Anhui Medical University, Hefei, China; ^3^National Infection Service, Public Health England, Bristol, United Kingdom; ^4^Bristol Medical School, University of Bristol, Bristol, United Kingdom

**Keywords:** antibiotic, respiratory tract infection, primary care, diagnosis, bacterial isolation

## Abstract

**Objectives:**

This paper aims to explore the direct associations of antibiotics prescription with clinical diagnosis and bacterial detection. It also analyses the relations of clinical diagnosis with symptoms and bacterial detection, with a hope of revealing indirect links to antibiotic prescription.

**Methods:**

The study was implemented in one village clinic and one township health center in each of four rural residential areas in Anhui Province, China. Observations were conducted to record clinical diagnosis and antibiotic prescription. A semi-structured questionnaire survey was used to collected patients' sociodemographic information and reported symptoms. Sputum and throat swabs were collected for bacterial culture.

**Results:**

Among 1,068 patients presenting in the study settings who received a diagnosis of respiratory tract infection (RTI), 87.8% of prescriptions included an antibiotic and 35.8% included two or more antibiotics. Symptomatic RTI patients to the site clinics were diagnosed mainly as having upper respiratory tract infection (32.0%), bronchitis/tracheitis (23.4%), others (16.6%), pharyngitis (11.1%), common cold (8.0%), pneumonia/bronchopneumonia (4.6%) and tonsillitis (4.3%). These clinical diagnosis were associated with symptoms to a varied degree especially for upper respiratory tract infection and bronchitis/tracheitis. Prescription of any antibiotics was positively associated with diagnosis of bronchitis/tracheitis (OR: 5.00, 95% CI: 2.63–9.51), tonsillitis (OR: 4.63, 95% CI: 1.48–14.46), pneumonia/bronchopneumonia (OR: 4.28, 95% CI: 1.40–13.04), pharyngitis (OR: 3.22, 95% CI: 1.57–6.59) and upper respiratory tract infection (OR: 3.04, 95% CI: 1.75–5.27). Prescription of two or more antibiotics was statistically significant related to diagnosis of bronchitis/ tracheitis (OR: 2.20, 95% CI: 1.44–3.35) or tonsillitis (OR: 2.97, 95% CI: 1.47–6.00). About 30% of the patients were identified with some type of bacteria. Bacteria detection was linked with pharyngitis (OR: 0.50, 95% CI: 0.28–0.88) but not prescription of antibiotics.

**Conclusions:**

Antibiotics prescription were found with a strong relation to diagnosis of RTIs given by the clinician but was not associated with the presence of bacteria in patient samples. Part of the diagnosis may have been given by the clinician to justify their antibiotics prescription. There is clear need to use additional measures (e.g., symptoms) in conjunction with diagnosis to supervise or audit excessive antibiotics use.

## Introduction

Antimicrobial resistance (AMR) is a global public health problem, which contributes to increased morbidity, mortality, and economic costs associated with infections (1, 2). AMR is caused primarily by over or inappropriate use of antibiotics (3–5). The bulk of human antibiotic use happens in primary care settings, with respiratory tract infections (RTI) accounting for over 80% of antibiotic prescriptions (6). The equivalent of primary care facilities in western countries, township health centers and village clinics in China, provide most outpatient care in rural areas, but antibiotic stewardship programs in these settings are much less developed than in higher level settings such as county, prefecture and provincial level hospitals (7, 8). Data from the National Center for Health Statistics of the United States shows that between 20 and 50% of outpatient antibiotic prescribing in the US is estimated to be unnecessary, which translates into nearly 47 million unnecessary antibiotic prescriptions each year (9). Excessive antibiotic use may be even more prevalent in China. Our previous study conducted in primary care settings of Anhui Province in China revealed that 88.0% of symptomatic RTI patients were prescribed antibiotics (6).

AMR containment depends heavily upon thorough understanding of drivers of antibiotic prescribing. Ideally, prescribing decisions should be evidence-based and information on bacterial presence together with antibiotic sensitivity can help. However, as in most countries, in China, microbiological tests are not available in primary care settings and patient samples are rarely sent to referral laboratories for testing (10). In consequence, existing studies in rural China have seldom investigated relationships between microbiological and clinical diagnoses in the treatment of RTI.

Another way of optimizing antibiotic prescribing is addressing clinical drivers. Several studies have found that symptoms reported by patients, diagnosis given by physicians and the social-demographic background of patients are all linked to antibiotic prescribing patterns (11–13). Other studies have reported that antibiotic prescribing rates are higher in rural (vs. urban) practices, among patients with longer illness duration or acute bronchitis, and when providers experience greater diagnostic uncertainty (14, 15). However, contemporary studies on determinants of antibiotic prescription in rural China suffer from two major shortcomings. First, they rely primarily on retrospective reports or review of patient records (16, 17). Retrospective reports are prone to biases, and this is especially true for rural residents many of whom are illiterate and may not be capable of distinguishing antibiotic from non-antibiotic medications; while in a previous study we found that electronic patient records do not match actual prescriptions to a large extent in rural China (18).

Funded jointly by the National Natural Science Foundation of China (NSFC) and UK Research and Innovation (UKRI) through the Newton Fund, we carried out a 3-year project titled “Pathways to optimizing antibiotic use in rural Anhui province, China” that aimed to investigate the magnitude and drivers of antibiotic use and antibiotic resistance in rural areas in China. The project adopted a mixed methodology making innovative use of non-participant observation, qualitative interviews, structured questionnaire surveys, microbiological testing and record review. The overall study protocol and results from other study components are published elsewhere (19, 20). This part of the study explores the direct associations of antibiotics prescription with clinical diagnosis and bacterial detection. It also analyses the relations of clinical diagnosis with symptoms and bacterial detection, with a hope of revealing indirect links to antibiotic prescription.

## Materials and Methods

### Recruitment Criteria

The study took place in one village clinic and one township health center in each of four counties in Anhui Province, China. Participants were male or female outpatients who were: (a) 18 years or older and able to give consent to participate in the study; (b) presenting to the recruitment site for the first time for the current illness; and (c) observed as having exacerbation of chronic obstructive pulmonary disease (COPD), upper respiratory tract infection with productive cough or sore throat.

These conditions were selected because they are common clinical presentations that can be associated with bacterial infection and where an organism may be identified through laboratory testing.

### Sampling and Sample Size

The clinics/centers in each county were selected randomly from a list provided by the provincial health board of all potential facilities fulfilling set criteria (population size, location, transport links, patient footfall). The participant patients were selected *via* a “consecutive sampling” in which, when a start date had been determined for a site, the recruitment continued daily (7 days a week) thereafter, until the target numbers had been reached. All presenting patients to the site village clinics and township health centers who met the inclusion criteria during any study day were invited to participate.

The sample size used for this study was calculated based on the microbiological sub-study aims. We estimate that at least 1,000 RTIs patients this will yield 100 *Streptococcus pneumoniae* isolates which should provide sufficient power to allow us to estimate key antibiotic susceptibilities. The detailed description and calculation of the sample size for this study has been reported in the published study protocol (19).

### Questionnaire and Data Collection Procedures

**Figure 1:**
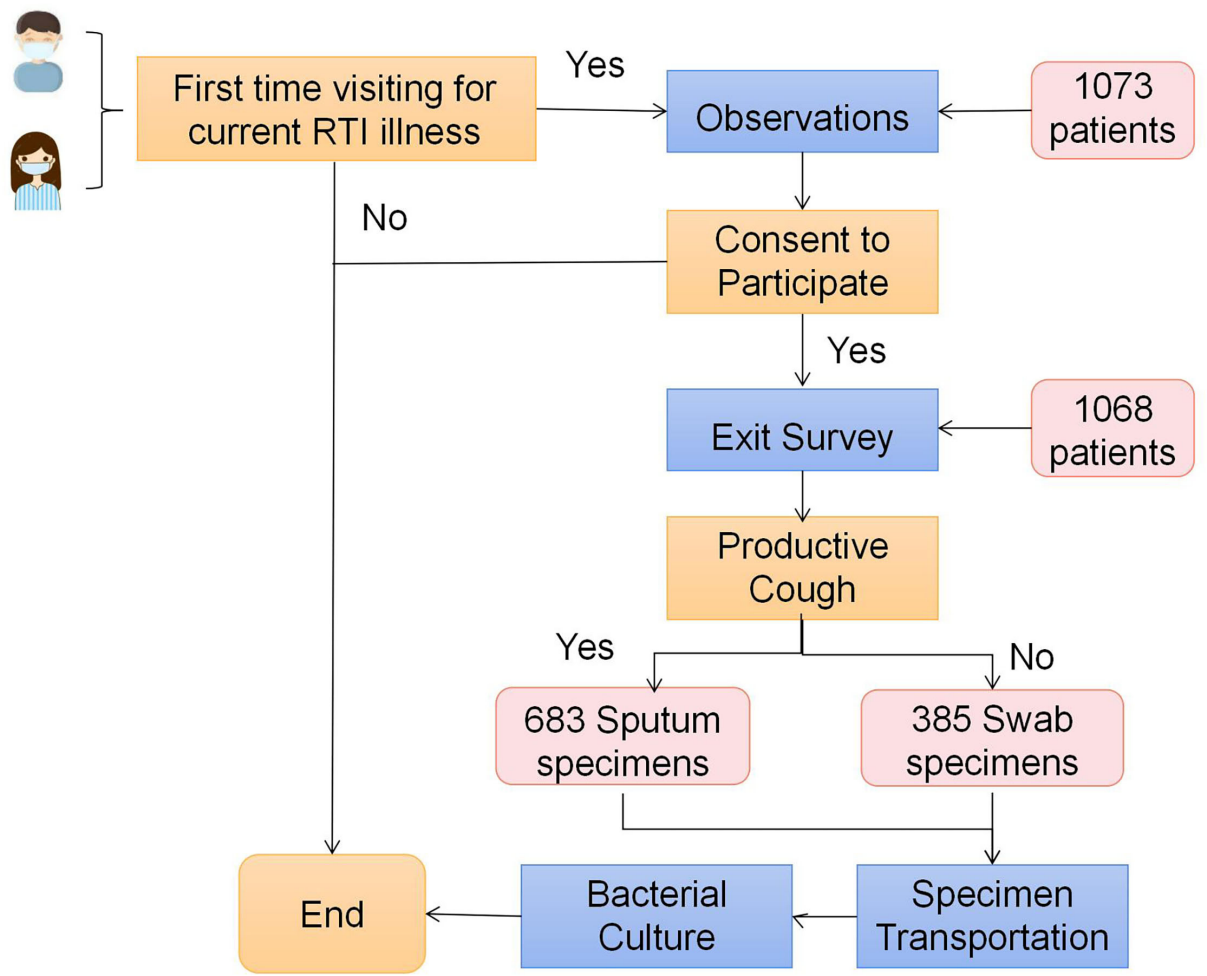
Flow chart of participant recruitment.

Data were collected from semi-structured observations, exit survey, specimen collection and testing (Figure 1). The observation focused on daily operational routine including test ordering, prescribing, patient recall and other standard procedures using a pre-designed worksheet (19). The exit survey was a brief face-to-face questionnaire consisting of structured and semi-structured questions and completed by all patients consented by the attending clinicians at clinics and health centers and recruited into the study. The questionnaire was informed by open-ended interviews undertaken in the study's pilot phase and included information on social demographics, symptoms and diseases history (Supplementary File 1). A trained researcher was sent to each participating clinics and health centers to perform semi-structured observations.

Sputum and throat swabs for bacterial culture, identification and susceptibility testing were collected. Sputum was collected from patients presenting with productive cough and throat swabs from patients with sore throat. The specimens were collected by the attending doctor using a sterilized container and according to a standard protocol. The specimens were transported and tested at the Central Laboratory of Anhui Medical University (AMU). For details are included in the published protocol (19).

### Data Management and Analysis

Questionnaire responses were double-entered into a database using EPI DATA 3.1, then exported and analyzed using SPSS. The analysis consisted of two parts. Part one centered on descriptive analysis using 2-sided x^2^, of null hypothesis, of the power of differences (*P* < 0.05) in the groups of education year (s), days since onset, symptoms, diagnosis, antibiotic use and bacterial detected between different sex and age groups.

Part two built 2 sets of multivariable logistic regression models aimed to derive the two kinds of associations as specified in the study purposes. More specifically, the first set of models used “any antibiotic prescription,” “combined antibiotic prescription,” and “bacterial isolation” as the dependent variable, respectively, and diagnosis, days since onset and social demographics as the independent variables. The second set of models used 7 categories diagnosis (including bronchitis/tracheitis, upper respiratory tract infection, pharyngitis, common cold, pneumonia/bronchopneumonia, tonsillitis, others diagnosis) as the dependent variable, respectively, and symptoms, days since onset and social demographics as the independent variables. Supplementary File 2 lists details of values assigned to variables used. The *P* (< 0.05) and OR value was used to judge whether a given variable is and to what extent linked to antibiotic prescription or bacterial isolation or clinical diagnosis. Any missing data were excluded from the analysis.

## Results

### Descriptive Characteristics of the Study Population

As shown in Table 1, a total of 1,068 patients aged 17–89 (51.0% males and 49.0% females) completed the exit survey and provided specimens, accounting for 99.5% of all the symptomatic RTI patients who met the inclusion criteria. Male and younger participants had more years of education than female and older ones (*P* = 0.000). Younger patients' visits to the clinics or health center showed a shorter time interval since onset of symptoms than older ones (*P* = 0.000). For the RTI patients, the proportion of antibiotic prescription, combined antibiotic prescription and bacterial detection was 87.8, 35.8, and 30.8%, respectively. Antibiotic prescription rate showed no statistical differences between sex and age subgroups, but men and older patients were more likely to get prescriptions containing two or more antibiotics than women and younger patients. There was a significant difference in the percentage of samples from which a bacterial pathogen was isolated by sex [higher in males (33.8%) than females (27.7%), *P* = 0.033] and age [older (39.6%) than younger (23.5%) patients, *P* = 0.001].

**Table 1:** Descriptive statistics of antibiotics prescription, symptoms, diagnosis and common socio-demographics, *N* (%).

	**Sex**		***P***	**Age**				***P***	**Total**
	**Male**	**Female**		**≤39**	**40–53**	**54–64**	**≥65**		
**Year(s) of education**
0	89 (16.3)	187 (35.8)	0.000	2 (0.7)	53 (18.7)	101 (40.1)	120 (46.2)	0.000	276 (26.0)
1–5	157 (28.8)	149 (28.5)		32 (11.8)	115 (40.5)	72 (28.6)	87 (33.5)		306 (28.8)
6–8	155 (28.4)	74 (14.1)		68 (25.0)	82 (28.9)	45 (17.9)	34 (13.1)		229 (21.6)
>8	141 (25.9)	109 (20.8)		167 (61.4)	33 (11.6)	33 (13.1)	17 (6.5)		250 (23.6)
Missing	3 (0.6)	4 (0.8)		3 (1.1)	1 (0.4)	1 (0.4)	2 (0.8)		
**Days since onset**
≤2 days	163 (29.9)	171 (32.7)	0.758	102 (37.5)	98 (34.5)	65 (25.8)	69 (26.5)	0.000	334 (31.5)
2–3.5 days	155 (28.4)	137 (26.2)		85 (31.3)	84 (29.6)	59 (23.4)	64 (24.6)		292 (27.5)
3.5–7 days	118 (21.7)	112 (21.4)		50 (18.4)	58 (20.4)	63 (25.0)	59 (22.7)		230 (21.7)
>7 days	105 (19.3)	99 (18.9)		33 (12.1)	43 (15.1)	62 (24.6)	66 (25.4)		204 (19.2)
Missing	4 (0.7)	4 (0.8)		2 (0.7)	1 (0.4)	3 (1.2)	2 (0.8)		
**Symptoms**
Blocked nose	118 (21.7)	139 (26.6)	0.060	95 (34.9)	72 (25.4)	53 (21.0)	37 (14.2)	0.000	257 (24.1)
Runny nose	140 (25.7)	137 (26.2)	0.850	62 (22.8)	69 (24.3)	63 (25.0)	83 (31.9)	0.080	277 (25.9)
Snotty nose	25 (4.6)	45 (8.6)	0.008	20 (7.4)	20 (7.0)	17 (6.7)	13 (5.0)	0.698	70 (6.6)
Dry cough	59 (10.8)	78 (14.9)	0.046	45 (16.5)	43 (15.1)	28 (11.1)	21 (8.1)	0.014	137 (12.8)
Cough with green sputum	120 (22.0)	130 (24.9)	0.273	82 (30.1)	70 (24.6)	61 (24.2)	37 (14.2)	0.000	250 (23.4)
Cough with white sputum	302 (55.4)	223 (42.6)	0.000	92 (33.8)	120 (42.3)	135 (53.6)	178 (68.5)	0.000	525 (49.2)
Dry/burning throat	120 (22.0)	138 (26.4)	0.096	70 (25.7)	79 (27.8)	51 (20.2)	58 (22.3)	0.169	258 (24.2)
Itchy throat	148 (27.2)	110 (21.0)	0.019	71 (26.1)	73 (25.9)	54 (21.4)	60 (23.1)	0.547	258 (24.2)
Sore throat	236 (43.3)	325 (62.1)	0.000	182 (66.9)	157 (55.3)	121 (48.0)	101 (38.8)	0.000	561 (52.5)
Breathing difficulties	179 (32.8)	186 (35.6)	0.349	59 (21.7)	84 (29.6)	95 (37.7)	127 (48.8)	0.000	365 (34.2)
Headache	84 (15.4)	117 (22.4)	0.004	67 (24.6)	48 (16.9)	48 (19.0)	38 (14.6)	0.021	201 (18.8)
Weakness	65 (11.9)	74 (14.1)	0.281	40 (14.7)	37 (13.0)	31 (12.3)	31 (11.9)	0.783	139 (13.0)
Fever	77 (14.1)	69 (13.2)	0.656	47 (17.3)	36 (12.7)	30 (11.9)	33 (12.7)	0.249	146 (13.7)
Other symptoms	98 (18.0)	115 (22.0)	0.101	45 (16.5)	53 (18.7)	59 (23.4)	56 (21.5)	0.206	213 (19.9)
**Diagnosis**
D1	144 (26.4)	106 (20.3)	0.018	36 (13.2)	62 (21.8)	66 (26.2)	86 (33.1)	0.000	250 (23.4)
D2	166 (30.5)	176 (33.7)	0.264	98 (36.0)	99 (34.9)	91 (36.1)	54 (20.8)	0.000	342 (32.0)
D3	47 (8.6)	72 (13.8)	0.008	51 (18.8)	40 (14.2)	17 (6.7)	11 (4.2)	0.000	119 (11.1)
D4	48 (8.8)	37 (7.1)	0.296	18 (6.6)	25 (8.8)	16 (6.3)	26 (10.0)	0.346	85 (8.0)
D5	26 (4.8)	23 (4.4)	0.771	4 (1.5)	7 (2.5)	19 (7.5)	19 (7.3)	0.000	49 (4.6)
D6	22 (4.0)	24 (4.6)	0.657	25 (9.2)	12 (4.2)	4 (1.6)	5 (1.9)	0.000	46 (4.3)
D7	92 (16.9)	85 (16.3)	0.783	40 (14.7)	39 (13.7)	39 (15.5)	59 (22.7)	0.022	177 (16.6)
Antibiotic use	483 (88.6)	455 (87.0)	0.417	232 (85.3)	255 (89.8)	219 (86.9)	232 (89.2)	0.343	938 (87.8)
Combined antibiotic use	219 (40.2)	163 (31.2)	0.002	64 (23.5)	107 (37.7)	102 (40.5)	109 (41.9)	0.000	382 (35.8)
Bacterial detected	184 (33.8)	145 (27.7)	0.033	64 (23.5)	81 (28.5)	81 (32.1)	103 (39.6)	0.001	329 (30.8)
**Total**	545 (51.0)	523 (49.0)		272 (25.5)	284 (26.6)	252 (23.6)	260 (24.3)		1,068

*D1, Bronchitis/tracheitis; D2, upper respiratory tract infection; D3, Pharyngitis; D4, Common cold; D5, Pneumonia/bronchopneumonia; D6, Tonsillitis; D7, Others*.

Among all the patients, the most frequently reported symptoms were sore throat (561, 52.5%), followed by cough with white sputum (525, 49.2%) and breathing difficulties (365, 34.2%). Compared with female patients, male patients were less likely to report snotty nose (4.6 vs. 8.6%), dry cough (10.8 vs. 14.9%), sore throat (43.3 vs. 62.1%) and headache (15.4 vs. 22.4%) but more likely to report cough with white sputum (55.4 vs. 42.6%) and itchy throat (27.2 vs. 21.0%). Compared with older patients, younger ones were more likely to report blocked nose, dry cough, cough with green sputum, sore throat and headache, while being less likely to report cough with white sputum and breathing difficulties. The top diagnoses given by the attending doctors were upper respiratory tract infection (342, 32.0%) and, bronchitis/tracheitis (250, 23.4%).

### Factors Associated With Antibiotic Prescription

Model 1 and Model 2 in Table 2 describes the statistics from the logistic regression modeling of “any antibiotic prescription” and “combined antibiotic prescription”. Any antibiotic prescription was positively associated with diagnosis of bronchitis/tracheitis (OR: 5.00, 95% CI: 2.63–9.51), tonsillitis (OR: 4.63, 95% CI: 1.48–14.46), RTI (OR: 3.04, 95% CI: 1.75–5.27), pharyngitis (OR: 3.22, 95% CI: 1.57–6.59) and pneumonia/bronchopneumonia (OR: 4.28, 95% CI: 1.40–13.04) but negatively linked with patients reporting illness duration of over seven days as compared with those reporting an illness duration of 2 days or less (OR: 0.27, 95% CI: 0.16–0.46). Combined antibiotic prescription was more likely to be given to patients aged 40 years older and in patients diagnosed with bronchitis/tracheitis (OR: 2.20, 95% CI: 1.44–3.35) or tonsillitis (OR: 2.97, 95% CI: 1.47–6.00). But less likely to be given to patients reporting longer illness duration (more than 7 days) (OR: 0.62, 95% CI: 0.41–0.94).

**Table 2:** Multivariable logistic regression statistics between diagnosis and antibiotic prescription or bacterial isolation.

**Independent variables**	**Model1: any antibiotic prescription**				**Model2: combined antibiotic prescription**				**Model3: bacterial detected**			
	**OR**	**95% C.I**		***P***	**OR**	**95% C.I**		***P***	**OR**	**95% C.I**		***P***
		**Lower**	**Upper**			**Lower**	**Upper**			**Lower**	**Upper**	
Sex (female as ref.)	0.80	0.52	1.23	0.300	0.70	0.52	0.94	0.017	0.78	0.58	1.05	0.100
**Age**												
≤39	**Ref**.		0.632	**Ref**.	0.008	**Ref**.	0.191					
40–53	1.26	0.67	2.35	0.471	1.94	1.26	3.00	0.003	1.33	0.86	2.07	0.200
54–64	1.01	0.52	1.93	0.985	2.09	1.32	3.31	0.002	1.34	0.84	2.14	0.214
≥65	1.42	0.69	2.94	0.346	2.11	1.29	3.45	0.003	1.72	1.05	2.81	0.031
**Year (s) of education**												
0	**Ref**.		0.215	**Ref**.	0.812	**Ref**.	0.382					
1–5	0.88	0.49	1.57	0.669	1.05	0.72	1.51	0.815	0.74	0.51	1.07	0.106
6–8	1.02	0.51	2.05	0.946	1.04	0.67	1.60	0.867	0.78	0.50	1.20	0.253
≥9	0.56	0.28	1.12	0.102	0.86	0.53	1.39	0.537	0.89	0.55	1.45	0.646
**Days since onset**												
≤2 days	**Ref**.		0.000	**Ref**.	0.064	**Ref**.	0.024					
2–3.5 days	0.80	0.46	1.39	0.429	1.00	0.71	1.42	0.991	1.43	0.99	2.05	0.054
3.5–7 days	1.24	0.65	2.38	0.518	1.03	0.71	1.51	0.861	1.55	1.05	2.27	0.027
>7 days	0.27	0.16	0.46	0.000	0.62	0.41	0.94	0.023	1.83	1.22	2.74	0.004
Bacterial detected/antibiotic prescription	1.17	0.75	1.81	0.487	0.92	0.69	1.23	0.590	1.19	0.77	1.84	0.437
**Diagnosis**												
Bronchitis/tracheitis	5.00	2.63	9.51	0.000	2.20	1.44	3.35	0.000	0.73	0.48	1.11	0.136
Upper respiratory tract infection	3.04	1.75	5.27	0.000	1.46	0.96	2.21	0.079	0.71	0.47	1.07	0.100
Pharyngitis	3.22	1.57	6.59	0.001	0.58	0.31	1.05	0.073	0.50	0.28	0.88	0.016
Common cold	1.09	0.55	2.16	0.799	0.35	0.17	0.71	0.004	0.64	0.36	1.15	0.138
Pneumonia/bronchopneumonia	4.28	1.40	13.04	0.011	1.81	0.93	3.52	0.081	0.97	0.50	1.88	0.931
Tonsillitis	4.63	1.48	14.46	0.008	2.97	1.47	6.00	0.002	0.96	0.47	1.98	0.914
Constant	6.33				0.46				0.48			

### Factors Associated With Bacterial Isolation

Model 3 in Table 2 provides the statistics from the logistic regression modeling of “bacterial detected”. The likelihood of detecting a bacterial isolate was higher in biological samples from patients aged 65 years or older than from those under 40 years (OR: 1.72, 95% CI: 1.05–2.81) and also from patients who presented at the health facility 3.5–7 days (OR: 1.55, 95% CI: 1.05–2.77) or more than 7 days (OR: 1.83, 95% CI: 1.22–2.74) since onset of infection than from those who presented within 2 days but was lower in patients diagnosed with pharyngitis (OR: 0.50, 95% CI: 0.28–0.88). No statistical association was observed between bacteria detection and prescription of antibiotics.

### Relationships Between Symptoms and Diagnosis

Table 3 summarizes results from the logistic regression analysis undertaken to explore possible symptom determinants for the range of clinical diagnoses assigned by the participating doctors in our study. After controlling for sex, age and education, diagnosis of “bronchitis/tracheitis” was found to be positively linked to symptoms of breathing difficulties (OR: 1.94, 95% CI: 1.40–2.68) and to a longer duration (3.5–7 days) since onset of infection compared with ≤2 days (OR: 2.51, 95% CI: 1.62–3.89), while negatively linked to sore throat (OR: 0.53, 95% CI: 0.38–0.75). “RTI” witnessed a positive association with blocked nose (OR: 1.68, 95% CI: 1.22–2.32), runny nose (OR: 1.57, 95% CI: 1.15–2.15), snotty nose (OR: 1.86, 95% CI: 1.07–3.23), sore throat (OR: 1.40, 95% CI: 1.04–1.88) and headache (OR: 1.48, 95% CI: 1.03–2.12), while a negative association with breathing difficulties (OR: 0.63, 95% CI: 0.46–0.87) and days since infection onset (vs. ≤2 days). “Common cold” showed positive link with blocked nose (OR: 2.29, 95% CI: 1.37–3.83), runny nose (OR: 1.90, 95% CI: 1.17–3.11), cough with white sputum (OR: 2.22, 95% CI: 1.21–4.08) and itchy throat (OR: 1.83, 95% CI: 1.10–3.04), while negative link with breathing difficulties (OR: 0.29, 95% CI: 0.16–0.54) and > 7 days since onset (OR: 0.36, 95% CI: 0.15–0.86). Patients with other diagnoses were most frequently among patients with cough with green sputum (OR: 2.49, 95% CI: 1.61–3.86) and with longer duration of symptoms (>3.5 days).

**Table 3:** Multivariable logistic regression statistics between diagnosis and common factors.

	**D1**		**D2**		**D3**		**D4**		**D5**		**D6**		**D7**	
	**OR**	**95% C.I**.	**OR**	**95% C.I**.	**OR**	**95% C.I**.	**OR**	**95% C.I**.	**OR**	**95% C.I**.	**OR**	**95% C.I**.	**OR**	**95% C.I**.
Sex (female as ref.)	0.85	(0.60, 1.21)	0.91	(0.67, 1.23)	1.53	(0.96, 2.42)	0.97	(0.57, 1.65)	1.33	(0.65, 2.70)	1.12	(0.56, 2.24)	1.02	(0.69, 1.50)
**Age**
≤39	Ref.	Ref.	Ref.	Ref.	Ref.	Ref.	Ref.							
40–53	1.49	(0.88, 2.52)	1.20	(0.78, 1.84)	0.61	(0.34, 1.10)	1.72	(0.83, 3.60)	1.15	(0.30, 4.41)	0.68	(0.29, 1.63)	0.74	(0.42, 1.30)
54–64	1.49	(0.86, 2.58)	1.53	(0.96, 2.43)	0.34*	(0.17, 0.72)	1.30	(0.57, 2.93)	3.41	(0.96, 12.06)	0.39	(0.11, 1.35)	0.70	(0.38, 1.29)
≥65	1.66	(0.92, 3.00)	0.76	(0.45, 1.30)	0.20*	(0.08, 0.48)	2.31	(0.99, 5.38)	2.85	(0.74, 11.02)	0.59	(0.17, 2.10)	1.28	(0.67, 2.43)
**Year (s) of education**
0	Ref.	Ref.	Ref.	Ref.	Ref.	Ref.	Ref.							
1–5	0.72	(0.47, 1.10)	0.97	(0.65, 1.45)	2.20*	(1.10, 4.42)	0.76	(0.38, 1.51)	1.19	(0.56, 2.53)	1.11	(0.29, 4.29)	1.00	(0.62, 1.61)
6–8	0.96	(0.59, 1.57)	0.91	(0.57, 1.45)	1.14	(0.50, 2.60)	1.26	(0.58, 2.73)	0.91	(0.31, 2.65)	2.63	(0.71, 9.77)	0.93	(0.52, 1.65)
≥9	0.84	(0.48, 1.45)	0.94	(0.57, 1.57)	1.69	(0.73, 3.92)	1.16	(0.51, 2.63)	0.63	(0.19, 2.13)	2.79	(0.71, 10.92)	0.70	(0.37, 1.34)
**Days since onset**
≤2 days	Ref.	Ref.	Ref.	Ref.	Ref.	Ref.	Ref.							
2–3.5 days	1.41	(0.91, 2.19)	0.68*	(0.48, 0.96)	1.11	(0.65, 1.89)	0.87	(0.49, 1.55)	1.86	(0.76, 4.58)	1.18	(0.55, 2.53)	1.21	(0.72, 2.04)
3.5–7 days	2.51*	(1.62, 3.89)	0.48*	(0.33, 0.72)	0.61	(0.31, 1.19)	0.70	(0.37, 1.35)	1.10	(0.40, 3.01)	0.26	(0.06, 1.18)	2.09*	(1.25, 3.52)
>7 days	1.56	(0.98, 2.49)	0.27*	(0.17, 0.42)	1.38	(0.75, 2.55)	0.36*	(0.15, 0.86)	1.83	(0.72, 4.61)	1.71	(0.70, 4.19)	3.17*	(1.89, 5.33)
Blocked nose	0.72	(0.48, 1.07)	1.68*	(1.22, 2.32)	0.37*	(0.20, 0.68)	2.29*	(1.37, 3.83)	0.41	(0.16, 1.04)	0.50	(0.21, 1.18)	1.09	(0.72, 1.64)
Runny nose	0.99	(0.70, 1.41)	1.57*	(1.15, 2.15)	0.40*	(0.22, 0.72)	1.90*	(1.17, 3.11)	0.45	(0.20, 1.01)	0.87	(0.39, 1.95)	0.73	(0.48, 1.11)
Snotty nose	0.96	(0.52, 1.77)	1.86*	(1.07, 3.23)	0.31	(0.09, 1.07)	0.24	(0.03, 1.82)	1.24	(0.42, 3.65)	1.41	(0.37, 5.45)	0.98	(0.52, 1.87)
Dry cough	1.02	(0.58, 1.79)	1.46	(0.93, 2.31)	1.00	(0.55, 1.81)	0.52	(0.17, 1.64)	/	/	0.88	(0.40, 1.96)	0.88	(0.44, 1.76)
Cough with green sputum	0.74	(0.48, 1.15)	1.07	(0.74, 1.55)	0.39*	(0.21, 0.73)	1.67	(0.88, 3.18)	1.88	(0.85, 4.15)	0.17*	(0.05, 0.57)	2.49*	(1.61, 3.86)
Cough with white sputum	1.37	(0.92, 2.02)	0.83	(0.59, 1.17)	0.56*	(0.33, 0.95)	2.22*	(1.21, 4.08)	1.55	(0.72, 3.37)	0.21*	(0.08, 0.52)	1.40	(0.91, 2.14)
Dry/burning throat	0.81	(0.56, 1.18)	0.87	(0.63, 1.22)	1.39	(0.87, 2.21)	0.87	(0.49, 1.55)	1.11	(0.55, 2.22)	1.21	(0.59, 2.45)	1.08	(0.72, 1.61)
Itchy throat	0.93	(0.65, 1.34)	0.87	(0.62, 1.23)	0.85	(0.50, 1.44)	1.83*	(1.10, 3.04)	1.27	(0.61, 2.62)	1.71	(0.77, 3.78)	0.92	(0.60, 1.40)
Sore throat	0.53*	(0.38, 0.75)	1.40*	(1.04, 1.88)	1.33	(0.84, 2.12)	0.96	(0.58, 1.59)	0.77	(0.39, 1.50)	2.62*	(1.18, 5.84)	1.00	(0.68, 1.45)
Breathing difficulties	1.94*	(1.40, 2.68)	0.63*	(0.46, 0.87)	0.85	(0.51, 1.42)	0.29*	(0.16, 0.54)	1.65	(0.87, 3.11)	0.74	(0.32, 1.74)	1.24	(0.86, 1.80)
Headache	0.96	(0.62, 1.50)	1.48*	(1.03, 2.12)	0.68	(0.36, 1.27)	1.19	(0.63, 2.25)	0.21*	(0.06, 0.78)	0.69	(0.28, 1.66)	1.01	(0.63, 1.62)
Weakness	0.77	(0.47, 1.27)	1.06	(0.69, 1.60)	0.50	(0.23, 1.08)	1.02	(0.49, 2.14)	1.79	(0.77, 4.20)	1.03	(0.41, 2.62)	1.58	(0.97, 2.56)
Fever	0.93	(0.56, 1.55)	0.92	(0.61, 1.40)	0.71	(0.34, 1.45)	0.63	(0.27, 1.44)	2.59	(1.08, 6.25)	1.28	(0.53, 3.08)	1.42	(0.84, 2.39)
Constant	0.20		0.55		0.28		0.04		0.01		0.04		0.08	

*D1, Bronchitis/tracheitis; D2, upper respiratory tract infection; D3, Pharyngitis; D4, Common cold; D5, Pneumonia/bronchopneumonia; D6, Tonsillitis; D7, Others*.

## Discussion

The study found that 87.8% of prescriptions for patients presenting with symptoms associated with RTIs contained an antibiotic, which is substantially higher than that found at similar settings in the United States (14.7%), Africa region (46.8%), the United Kingdom (42.0%) and that recommended by the World Health Organization (15). In addition, more than one in three (35.8%) of prescriptions contained two or more antibiotics. These results suggest that excessive antibiotic use in primary health care settings in rural China is still very prevalent and there is a clear need for a better understanding of the issue.

Our study revealed that most of the clinical diagnoses except common cold were strong predictors of antibiotics use. Treatment should be based on diagnosis, however, the study shows that the antibiotic prescriptions were not necessarily based on the etiology/pathology. According to China national guidelines on rational medicine use for frontier medical care givers (21), antibiotics use is recommended only for pneumonia/bronchopneumonia but not for bronchitis/tracheitis, upper respiratory tract infection, pharyngitis and tonsillitis unless there are clear indications of bacterial infection. However, in our study, a diagnosis of any of these RTIs was a strong predictors of antibiotic prescription. Studies have documented only marginal effects of antibiotic use for these diagnoses, resulting in above a half-day reduction in cough but no reduction of functional impairment compared to placebo treatment (22). For some physicians, diagnosis was given for satisfying record keeping requirement rather than clinical purposes.

Prescription of combined (two or more) antibiotics demonstrated different relationships with diagnosis and symptoms. In terms of diagnosis, it was only positively linked to tonsillitis and bronchitis/tracheitis but negatively related to common cold. These findings may partly be explained, according to our qualitative interviews, by the beliefs that: (a) “common cold is generally, as indicated by its name, common, mild and self-limiting” and therefore “does not need antibiotics treatment”; (b) tonsillitis and bronchitis/tracheitis are “often caused by and/or accompanied with bacterial infections,” “are difficult to treat” and thus “need adequate, potent and broad-spectrum antibiotics”.

The evidence shows that the relationship between diagnosis and symptoms were consistent with national guidelines (21). However, a substantial proportion of patients (13.5%) were not given any diagnosis. This may be explained by the fact that it is difficult to reach a precise RTI diagnosis, especially in primary care settings where lab tests and sophisticated examinations are generally lacking and that consequently, doctors may be accustomed to providing presumptive treatment in the face of diagnostic uncertainty (23). One of the strengths of this study is the collection of clinical samples. However, bacterial detection was negatively linked only to the diagnostic category of pharyngitis. Another interesting and counterintuitive finding relates to duration of illness. Patients with longer than 3.5 days of illness duration were more likely to provide samples which produced positive bacterial cultures but less likely to be prescribed with antibiotics. This suggests that antibiotics may sometimes have been used as a “preventive” action rather than to treat existing bacterial infection or even simply to meet perceived demand since symptoms in the earlier the stage of RTI, can be more extensive thus the greater the perceived demand for a “quick cure” (24, 25). In addition, patients aged 65+ were more likely to provide samples from which bacteria could be grown. This may be partly attributable to reduced immunity and more comorbidities in the elderly population who are therefore, more prone to bacterial infection (26, 27). Finally, it is important to note that bacteria isolation was not associated with prescription of antibiotics.

### Implications for Research and Practice

Our study not only calls for additional attention to the excessive use of antibiotics in rural China but also sheds new lights on how to better understand and address the problem. In particular, future efforts should include: (a) research into determinants of antibiotics use for specific diagnoses, especially bronchitis/tracheitis, upper respiratory tract infection, pharyngitis and tonsillitis; (b) training of primary care doctors on compliance with management guidelines of commonly diagnosed RTIs, misapprehensions about these infections and the effectiveness of antibiotics in treating these infections, understanding patients' real demand and reassuring patients without antibiotics; (c) introducing microbiological tests into rural primary care in China and leveraging the test results into rational use of antibiotics, for example, using the results of regular microbiological surveillance surveys to inform local selection of narrow spectrum antibiotics or to reassure patients about the safety of not using an antibiotic; (d) educating patients/residents about disbenefits of unnecessary antibiotics and about clearly communicating their expectations of the consultation.

### Strengths and Limitations

This study has both strengths and limitations. It is the first study to collect data from healthcare providers and users through direct (non-participative) observation, whilst most existing research on antibiotic use in China relies on data from medical records or reports by health care professionals, who may be inclined to omit recording overuse or misuse of antibiotics so as to meet relevant policy requirements. It is also the first study to perform both microbiological testing and clinical data collection in rural and township care settings, thus enabling cross-linking of data from different sources. However, the study covered only either sites (village clinics or health centers) within a single province, so caution is warranted in generalizing our findings to other parts of China, although the social, cultural and economic background of Anhui is similar to the majority of areas in the nation. The use of observational methods may also have influenced, to some extent, the routine encounters between the patients and doctors and the prescription behaviors being observed, although we instituted a 2-week preparation period for each site clinic before starting data collection to allow the field researchers to build trust with the doctors and the research team is confident that doctors' prescribing practices were not unduly affected by their presence.

### Conclusions

Excessive use of antibiotics is still prevalent in rural Anhui, China. Most of the commonly diagnosed RTIs (bronchitis/tracheitis, pneumonia/bronchopneumonia, tonsillitis, pharyngitis and upper respiratory tract infection) were strong predictors of antibiotic prescription but common cold was not. Prescribing behavior was not associated with microbiological detection of bacteria in patient samples. Part of the diagnosis may have been given by the clinician to justify their antibiotics prescription. Therefore, there is clear need to use additional measures (e.g., symptoms) in conjunction with diagnosis to supervise or audit excessive antibiotics use.

## Data Availability Statement

The original contributions presented in the study are included in the article/Supplementary Material, further inquiries can be directed to the corresponding author/s.

## Ethics Statement

The studies involving human participants were reviewed and approved by Biomedical Ethics Committee of Anhui Medical University (reference number: 20170271). The patients/participants provided their written informed consent to participate in this study.

## Author Contributions

SX directed study implementation and data collection, conducted data analysis, and drafted the manuscript. DW and HL conceptualized and supervised the study and revised the manuscript together with IO. FR, ChaJ, and CheJ developed data collection materials and collected data together with SX. All authors contributed to the article and approved the submitted version.

## Funding

This study was supported by the Newton Fund [UK Research and Innovation (UKRI)] and the National Natural Science Foundation of China (NSFC, Grant Number 81661138001 and 81861138049) under the UK-China Antimicrobial resistance Partnership Initiative, Grant Number MR/P00756/1.

## Conflict of Interest

The authors declare that the research was conducted in the absence of any commercial or financial relationships that could be construed as a potential conflict of interest.

## Publisher's Note

All claims expressed in this article are solely those of the authors and do not necessarily represent those of their affiliated organizations, or those of the publisher, the editors and the reviewers. Any product that may be evaluated in this article, or claim that may be made by its manufacturer, is not guaranteed or endorsed by the publisher.
