# Supplementary material for: Relationships Between Diagnosis, Bacterial Isolation, and Antibiotic Prescription in Out Patients With Respiratory Tract Infection Symptoms in Rural Anhui, China
Source: Front Public Health. 2022 Feb 9;10:810348. doi: 10.3389/fpubh.2022.810348 (PMC8864097; doi:10.3389/fpubh.2022.810348)
Supplement: Supplementary file 1 [file Data_Sheet_1.PDF]

Recruitment Proforma

Participant ID: ________________

Date completed: ________   **Name of field worker completing proforma: ________**

1. **Demographics** (Please tick *and* enter the age number)

**1.1 Gender:**	**1.2 Age**
1 ☐ Female	_______
2 ☐ Male	

2. **Health problem as reported by patient or person accompanying patient**

Write down exactly what the patient or their representative first tells the physician （**in their own words**）:

____

____

2.1 Write down any additional **respiratory symptoms or urinary symptoms** that the patient or their representative reports to the physician (if not already mentioned in 2 above)

2.1.1 Symptoms reported by the patient (**in their own words)**:

____

2.1.2 Symptoms reported by family members/carers accompanying the patient (**in their own words):**

____

3. Illness trajectory

Record any other details about the illness reported by patient and/or carers (e.g. when symptoms began, duration, previous episodes of the same problem). If no other details are provided, tick 'Not applicable'.

____

☐Not applicable

3.1. Record patient's report of any previous treatment for the current illness, such as medicines taken **(in their own words)**

____

☐ None

4. Physician's diagnosis

Record the physician's diagnosis or statement of what the health problem is as told to the patient, **in his/her own words.** If no diagnosis is stated, then tick **'not stated'.**

____

____

☐ Not stated

5. Tests ordered by physician and carried out by patient (please tick or write down)

☐Blood test   ☐Urine test   ☐X-Ray   ☐CT   ☐B Ultrasound   ☐ECG

Other (Please Specify) ______________________________   ☐None

6. Tests ordered by physician but not carried out by patient (please tick or write down)

☐Blood test ☐Urine test ☐X-Ray ☐CT ☐B Ultrasound ☐ECG ☐None

Other (Please Specify) ________________________________

7. **Treatment prescribed / recommended** as stated during the consultation and heard by researchers

7.1 What name or type of medicine(s) that was/were prescribed as told by the physician, if any? Names, duration and dosages of any prescribed medicines (including supplementary medicines and herbs):

____

7.2 Does the physician give any information to the patient about treatment?

☐No ☐Yes

If Yes, what information does the physician give the patient, verbally or by handwriting, about treatment (such as how to take medicine):

☐**Verbal**: ☐ Go to have IV ☐Take medicine orally ☐Drink more water ☐ Rest

☐ Don't drink alcohol ☐ Don't smoke or smoke less ☐Avoid spicy/irritating food

☐ Other ____

☐**Handwritten**: ____

8. Electronic patient record (E), paper record (P) or no record (N) If no record, make a verbal request to the physician for the details after the consultation and record them, tick **'V'.**
(*Note:* After receiving informed consent from the patient, go back to the doctor to request this information and complete 8.1 and 8.2, or take a photo of the electronic or paper record of the patient's visit).

8.1 Diagnosis (record exactly as stated in the electronic patient record, tick 'E'. If no electronic record is created, record exactly as stated in paper logbook, tick 'P'. If no record is created, tick 'N' for none, and if verbal record taken from the doctor, tick V)

☐E ☐P ☐ N ☐ V

____

8.2 Prescription (record exactly as stated in the electronic patient record, including dosage, frequency and duration, tick '**E**'. If no electronic record is created, record exactly as stated in paper logbook or prescription notepad, tick '**P**'. If no record is created, tick '**N**' for none).

☐ E ☐ P ☐N

8.2.1 Were any antibiotics prescribed?

☐ No ☐Yes

8.2.2 If Yes, first tick the method of drug administration; then specify the name, total quantity and dosage:

☐ IV Antibiotics prescribed:

**Tick if prescribed**	**Medicine Name**	**Specification**	**Total quantity**	**Dosage**
	Penicillin			
	Levofloxacin			
	Ampicillin			
	Clindamycin			
	Amikacin			
	Azithromycin			
	Metronidazole			
	Fosfomycin sodium			
	Amoxicillin			
	Erythromycin			
	Norfloxacin			
	Cefradine			
Add others				

☐ Oral antibiotics prescribed:

**Tick if prescribed**	**Antibiotics Name**	**Specification**	**Total quantity**	**Dosage**
	Amoxicillin			
	Erythromycin			
	Norfloxacin			
	Cefradine			
**Add others**				

8.2.3 Other medicines prescribed:

**Tick if prescribed**	**Medicine Name**	**Total quantity**	**Dosage**	**Mode of administration**
	Dexamethasone			
	Tetracaine			
	chlorpheniramine			
	Aminophylline			
	Ribavirin			
	Compound paracetamol amine capsules			
	Ambroxol			
	Roxithromycin			
	Asthma-calming and cough-stopping patch (*pingchuan zhike pian*)			
	Qing kailing(qinghai ling)			
	Galculus Bovis and Metronidazole Capsules (*rengong niuhuang jia xiaocuo jiaonang*:			
**Add others**				

9. Any further observation note of the consultation process (Researcher's observation):

____

____

Antibiotic Pathway Study

Patient Consent Record & Contact Details

Dear patient

We invite you to participate in the project named "The pathway study of antibiotic use in China" funded by the Research Councils UK and the National Natural Science Foundation of China. This study has been approved by the Ethics Committee of Anhui Medical University. In order to protect your rights, please read the following statement of consent.

Consent statement (and see overleaf)

1.	I understand what this study is about and how I am being asked to participate in it.	
2.	I understand that taking part in this research is voluntary and that I am free to leave the study at any time, without giving any reason, and without my medical care or rights or those of my family being affected.	
3.	I understand that any information or sample I provide will be anonymised by removing all identifying details and will be kept strictly confidential and used only for research purposes. After the study, the anonymised information will be made publicly available for potential further research, but it will not be possible to identify me from the data.	
4.	I agree to a sputum/throat swab/urine [as applicable] sample being taken & sent to the Anhui Medical University laboratory for testing. I understand that anonymised samples may be stored for use in future research.	
5.	I agree to be contacted for an in-depth interview.	
6.	I understand that with my agreement, the interview will be audio-recorded but no one except the research team will hear this recording. I am aware that anonymised quotes from the interview may be included in reports of the study findings.	
7.	My consent is based on the University of Bristol and Anhui Medical University complying with their duties and obligations under the Data Protection Act.	

I understand the above statements and am fully aware of the risks and benefits that may be incurred in this study. I voluntarily participate in this study.

Participant ID: ____________________________

Participant Contact Details

Address: _______________________________

Phone number1: ________________________

Phone number2: ________________________

Participant Consent (when the participant is not able to sign for themselves, the researcher will sign on their behalf to record their consent):

Name of Participant	Date	Signature
Name of Family member	Date	Signature
Name of Person taking consent	Date	Signature

If the patient refuses the above informed consent statement, ask why he/she chooses not to participate.

Reason for refusal: ____

This information is to be either given to patients or discussed with the patients:

Your involvement in the study will remain confidential and your study data will be anonymised. Your information will be given an identification number and any information that could identify you will be removed so it will not be possible to identify you in any way.

At the end of the study all the data will be made “Open Access” which means that it will be stored in an online database so that it is publicly available for future research. This information will only be available to other researchers and national bodies which monitor research studies and there will be no way to identify you from it.

Patient Exit Survey

Participant ID: __________________
Date completed: __________________

Name of field worker completing survey: _______________________________

*Please read out the questions below that are **highlighted in bold** only. Do not read out the response options, unless the question has a specific instruction to do so.*

1.1 Where do you live?

Name of town or village: ______________________________________

2 Patient-Informant Understanding of illness

2.1 What illness have you come about today?
____

2.2 How many days have you been feeling unwell?
__________days

2.3a Record below ongoing symptoms only
[Complete this section if patient reports respiratory tract symptoms in 2.1. If patient reports urinary tract symptoms, leave this section blank and go to 2.3b]

2.3a1 Do you have any nasal symptoms?
- ☐ No
- ☐ Yes

(If yes) **What are they?** *[Tick all applicable boxes]*
- ☐ Blocked nose
- ☐ Runny nose (clear/watery discharge)
- ☐ Snotty nose (yellow/green discharge)
- ☐ Other *[please specify]* ____

2.3a.2 Do you have a cough?
- ☐ No
- ☐ Yes

(If yes) **How would you describe it?** *[Tick all applicable boxes]*
- ☐ Dry cough
- ☐ Cough with green sputum
- ☐ Cough with white sputum
- ☐ Other *[please specify]* ____

2.3a.3 Do you have any throat problems?
- ☐ No
- ☐ Yes

(If yes) **How would you describe them?** *[Tick all applicable boxes]*
- ☐ Dry throat

- ☐ Sore throat
- ☐ Itchy throat
- ☐ Burning throat
- ☐ Rough/hoarse voice
- ☐ Difficulty swallowing
- ☐ Other *[please specify]* ___________________________________

2.3a.4 Do you have any breathing difficulties?

- ☐ No
- ☐ Yes

2.3a.5 In addition to these symptoms, do you have any other symptoms?

- ☐ No
- ☐ Yes

(If yes) **What are they?** *[prompt for each symptom and tick all applicable boxes]*

- ☐ Headache
- ☐ Weakness
- ☐ Fever
- ☐ Other *[please specify]* ____________________________________

<u>2.3b *(Complete this section if patient reports urinary tract symptoms in 2.1)*</u>

2.3b.1 What symptoms do you have related to UTI ? *[Tick all applicable boxes]*

- ☐ Urinary frequency
- ☐ Urinary urgency
- ☐ Pain/difficulty while urinating/dysuria
- ☐ Urinary incontinence
- ☐ Blood in urine/hematuria
- ☐ Turbid urine
- ☐ Lower back pain
- ☐ Urinary tract itching
- ☐ Lower stomach feeling heavy
- ☐ Other *[please specify]* ____

2.4 What treatment were you given? *[Tick all applicable boxes]*

- ☐ IV
- ☐ Oral medicine (pills)
- ☐ Chinese herbs
- ☐ Other

2.4.1 (If medicines were prescribed, including IV**) Do you know what it is for?**

- ☐ Yes
- ☐ No
- ☐ Not Stated

(If yes) **What it is for** *(do not probe the function of any specific medicine):*

____

2.4.2 *(If applicable)* **As far as you know, does this contain antibiotics or anti-inflammation medicine?**

☐ Yes
☐ No ☐ Not sure

2.4.3 *(If applicable)* **When will you stop using the medicines?**
☐ When symptoms start to relieve;
☐ When main symptoms have disappeared;
☐ When all symptoms have disappeared;
☐ When I have taken all the pills prescribed;
☐ When I feel almost recovered;
☐ Other (please specify)_______________________________________

2.4.4 If you have any medicines left over, what will you do with them?
☐ Throw them away
☐ Keep them at home for future use
☐ Keep them at home for me to use next time
☐ Keep them at home but throw away when past expiry date
☐ Give it to family or friends;
☐ Other (please specify)_______________________________________

2.5 Did you want any other treatment:
☐ *No*
☐ *Yes*
☐ *Not stated*

2.5.1 *(If yes)* **What was it?** *[Tick all applicable boxes]*
☐ IV medicines
☐ Oral medicines (pills)
☐ Herbs
☐ Acupuncture
☐ Anti-bacterial medicine
☐ Anti-inflammation medicine
☐ Other (please specify)_______________________________________

2.6 Have you used any self-treatment at home for this problem before coming here?
☐ No
☐ Yes
(If yes) **What was it?** *[Tick all applicable boxes]*
☐ Medicine for oral intake
☐ Medicine for plastering or pasting
☐ Herbs
☐ Anti-bacterial medicine
☐ Anti-inflammation medicine
☐ Drink warm water
☐ Rested more
☐ Other *[please specify]* _______________________________________

2.6.1 (If applicable**) As far as you know, did the pills contain antibiotics or anti-inflammation medicine?**
☐ Yes
☐ No ☐ Not sure

2.7 Did you get any treatment outside your home for this problem before coming here?

☐ No
☐ Yes

2.7.1 (If yes) **What was it?** *[Tick all applicable boxes]*

☐ IV
☐ Medicine for oral intake
☐ Herbs
☐ Anti-bacterial medicine
☐ Anti-inflammation medicine
☐ Other *[please specify]* ____

2.7.2 (If applicable) **As far as you know, did it contain antibiotics or anti-inflammation medicine?**

☐ Yes ☐ Not sure
☐ No

2.7.3 Where did you obtain these treatment? *[Tick all applicable boxes]*

☐ Pharmacy
☐ Township Health Center
☐ Village Clinic
☐ Private doctor
☐ From Neighbor or Friend
☐ Other *[please specify]*_________________

3.1 Have you had a similar illness in the past year? If yes, how many times?

☐ No
☐ Yes, ____times
☐ Many times, but can't remember the specific number

3.2 Have you taken medicines, including Western or Chinese, for these or any other health reasons in the past year?

☐ No (skip to 3.3)
☐ I can't remember (skip to 3.3)
☐ Yes, Western medicine
☐ Yes, Chinese medicine (skip to 3.3)
☐ Other *[please specify]* __________________________________

3.2.1 *[If Western medicines are mentioned]* **As far as you know, were any of these medicines anti-inflammatory?**

☐ Yes
☐ No (skip to 3.3)
☐ I don't know what antibiotics/anti-inflammatory medicine is (skip to 3.3)
☐ I don't remember what my medicines were (skip to 3.3)
☐ Not stated (skip to 3.3)
☐ Other *[please specify]*:

____

3.2.2 If you have used "anti-inflammation medicine", how many times have you used them in the past 12 months?

________ times

☐ I can't remember

3.2.3 [If yes] Where did you obtain these medicines?

☐ Hospital or Clinic Pharmacy
☐ Retail Pharmacy
☐ From Family Member
☐ From Neighbor or Friend
☐ I already have them at home
☐ Other *[please specify]* ________________

3.3 Have you heard of "drug resistance"?

☐ No
☐ Yes

4 Demographics

4.1 What is your year of birth? ________Y

4.2 How many years of education have you completed? *[Write down the exact number]*

________________years

4.3 If you have lived outside your home residence for more than a month over the past year, how many months have you been away?

________months

[If yes] **Is the place where you have been living urban or rural?**

☐ Urban
☐ Rural

4.4 What is your Household registration status (*hukou*) for accessing health care benefits?

☐ Urban
☐ Rural
☐ Other____________________

4.5 What type of insurance do you have, if any?

☐ New rural cooperative Medical insurance scheme
☐ Urban Employee's Medical Insurance
☐ Urban Residents' Medical Insurance
☐ Not Having Medical Insurance
☐ Other Type of Insurance *[please specify]*: __________________________
____

4.6 Can you tell me who paid for your visit today? *[Tick one applicable answer]*

- ☐ Myself
- ☐ Spouse
- ☐ Father
- ☐ Mother
- ☐ Daughter
- ☐ Son
- ☐ Grandparent
- ☐ Grandchild
- ☐ Other *[please specify]*: ______________________________________

Microbiological Tests

Specimen acceptance

1) Is the case properly sealed? ☐ Yes ☐No
2) Whether there is still ice inside the case? ☐Yes ☐No
3) Total tubes of specimens received:__________
4) Number of specimen tubes being properly capped:__________
5) Number of specimen tube being clearly labelled:__________
6) Name of Technician/student:__________
7) Date and time of acceptance (automatically recorded) __________

Specimen assessment

1) Reference number: ____________________
2) Type of specimen ☐Sputum ☐throat swab ☐urine
3) Condition check (this applies to sputum/throat swab/urine specimen)
   a) Do you identify any labelling problems with the specimen?
      ☐without label
      ☐with undiscernible label
      ☐with inconsistent label
      ☐others(please specify) ____________________

   b) Do you identify any indications of specimen contamination?
      ☐not properly capped
      ☐with dust on or in the specimen
      ☐others(please specify) ____________________
   c) Has the specimen exceeded pre-set time limit of preservation (i.e., 2 hours under room temperature and 8 hours under 4℃).
      ☐Yes ☐No

For specimen that has not passed condition check, the lab test ends.

4) Appearance examination (this applies only to sputum specimens that have passed the above condition check)
   a) Is the sputum qualified?
      ☐Yes, since it features:
         ☐Yellow/grey ☐bloody ☐rust ☐opacitas ☐lumpy
         ☐others(please specify) ____________________
      ☐No, since it
         ☐Is water-like ☐is saliva-like ☐contains food fragments ☐contains dusts
         ☐others(please specify) ____________________
5) Cellular observation (this applies only to the sputum specimens that have passed the above appearance examination)
   a) Is the sputum qualified?
      ☐Yes, since it has
         ☐>25 pus cells/LP ☐<10 squamous epithelium/LP
      ☐No, since it has
         ☐<10 pus cells /LP ☐bacteria count >+++ ☐>25 squamous epithelium/ LP

6) Name of Technician __________
7) Date and time of acceptance (automatically recorded)__________

Specimen pre-treatment

1) Reference number: __________
2) Starting time of specimen digestion __________

Reading of stained specimen

1) Reference number: __________
2) Starting time of stained specimen reading __________
3) Identification of engulfed bacteria? ☐Yes ☐No

   If yes, please describe the bacteria in terms arrangement and shape (e.g., thinness, length, twists).____

Specimen inoculation

1) Reference number: __________
2) (For urine specimen only) Correction values for the two inoculation rings:

   1μL ring:________

   10μL ring:_______
3) (For urine specimen only) Selection of inoculation ring:

   ☐1μL ring (if the patient has not used antibiotics)

   ☐10μL ring (if the patient has already used antibiotics)
4) Starting time of specimen inoculation

Manual bacteria identification

1) Reference number: __________
2) Date and time of identification__________
3) Identification of bacteria? ☐Yes ☐No

   If yes, please enter the results of manual colony identification:

   (a) Colony morphology____
   (b) Catalyst: ☐ Negative ☐ Positive
   (c) Coagulase: ☐ Negative ☐ Positive
   (d) Oxidase: ☐ Negative ☐ Positive
   (e) Factor V: ☐ Negative ☐ Positive
   (f) Factor X: ☐ Negative ☐ Positive
   (g) OP: ☐ Negative ☐ Positive
   (h) other____
4) Name of Technician __________

Automated Bacterial Detection

1) Reference number: __________

2) Date and time of identification___________

3) Name of Technician ___________

4) (If manual bacteria identification reveals colony growth) Please enter the name of bacteria

____

Susceptibility test results

1) Reference number ___________
2) Name of bacteria ___________
3) Date and time of susceptibility test ___________

Antibiotics	DISK	MIC	Interps
Amikacin			
Ampicillin			
Ampicillin / Shubatan			
Amoxicillin / clavulanate			
Aztreonam			
Cefazolin			
Cefepime			
Cefoperazone / Shubatan			
Cefoxitin			
Ceftazidime			
Cefuroxime			
Chloramphenicol			
Clindamycin			
Erythromycin			
Gentamicin (or tobramycin)			
Gentamicin or levofloxacin			
Imipenem			
Levofloxacin			
Linezolid			
Meropenem			
Minocycline			
Moxifloxacin			
Penicillin			
Piperacillin			
Piperacillin / tazobactam			
Polymyxin			
Rifampin			
SMZco			
Atreptomycin			
Teicoplanin			
Tigecycline			
Vancomycin			
**Add others**			
