# Supplementary material for: Relationships Between Diagnosis, Bacterial Isolation, and Antibiotic Prescription in Out Patients With Respiratory Tract Infection Symptoms in Rural Anhui, China
Source: Front Public Health. 2022 Feb 9;10:810348. doi: 10.3389/fpubh.2022.810348 (PMC8864097; doi:10.3389/fpubh.2022.810348)
Supplement: Supplementary file 2 [file Data_Sheet_2.PDF]

Supplementary file 2 Values assgined to the variables

Varables	Value	Variables	Value
**Sex**		**Diagnosis**	
Male	1	*Bronchitis/tracheitis(D1)*	Yes=1; No=0
Female	2	*Upper respiratory tract infection(D2)*	Yes=1; No=0
**Age**		*Pharyngitis(D3)*	Yes=1; No=0
⩽39	1	*Common cold(D4)*	Yes=1; No=0
40-53	2	*Pneumonia/bronchopneumonia(D5)*	Yes=1; No=0
54-64	3	*Tonsillitis(D6)*	Yes=1; No=0
⩾ 65	4	*Others(D7)*	Yes=1; No=0
**Year(s) of education**		**Symptoms**	
0	1	*Blocked nose*	Yes=1; No=0
1-5	2	*Runny nose*	Yes=1; No=0
6-8	3	*Snotty nose*	Yes=1; No=0
⩾ 9	4	*Dry cough*	Yes=1; No=0
**Days since onset**		*Cough with green sputum*	Yes=1; No=0
⩽ 2 days	1	*Cough with white sputum*	Yes=1; No=0
2-3.5 days	2	*Dry / burning throat*	Yes=1; No=0
3.5-7 days	3	*Itchy throat*	Yes=1; No=0
>7 days	4	*Sore throat*	Yes=1; No=0
Antibiotic use	Yes=1; No=0	*Breathing difficulties*	Yes=1; No=0
Combined antibiotic use	Yes=1; No=0	*Headache*	Yes=1; No=0
Bacterial detected	Yes=1; No=0	*Weakness*	Yes=1; No=0
		Fever	Yes=1; No=0
		Other Symptoms	Yes=1; No=0
